# Curcumin induces apoptosis and autophagy inhuman renal cell carcinoma cells via Akt/mTOR suppression

**DOI:** 10.1080/21655979.2021.1960765

**Published:** 2021-08-17

**Authors:** Xuelian Gong, Ling Jiang, Wei Li, Qingbin Liang, Zhen Li

**Affiliations:** a Department of Pharmacy, Qishan Hospital, Yantai, China; b Department of Emergency, Qingdao Women and Children’s Hospital, Yantai, Shandong, China; c Yantai Yuhuangding Hospital, Yantai, Shandong, China

**Keywords:** Renal cell carcinoma (RCC), curcumin, apoptosis, carcinoma, autophagy

## Abstract

Renal cell carcinoma (RCC) is a highly aggressive cancer leading to high economic and social burden, and has increasing annual cases. Curcumin is a traditional Chinese medicine widely used as anti-inflammatory, anti-viral and anti-cancer agent, thus can be applicable in RCC therapy. The work assessed the effects of RCC treatment with Curcumin, Curcumin+3-MA, Curcumin+ CQ or curcumin+ Z-VAD in vitro and in vivo, and the mechanisms involved in inhibition of tumor cells proliferation. The study used ACHN tumor cells and C57BL/6 nude mice for results validation. Cell proliferation was determined through MTT assays while apoptosis was investigated using Annexin V-FITC/PI kit and flow cytometry. Enzyme-linked immunosorbent assay (ELISA) was used to detect IL-6, IL-8, and TNF-α cytokines expressions. AKT/mTOR and autophagy proteins expressions were investigated through western blot and immunofluorescence. The results indicated significantly inhibited cell viability following ACHN tumor cells treatments with curcumin alone, or with the various combinations, as compared to the control. Apoptosis was significantly increased following curcumin treatment, but was significantly reversed after treatment with curcumin+ 3-MA. Likewise, AKT/mTOR proteins expression were significantly reduced while the autophagy-related proteins were significantly elevated following curcumin treatment. The tumor size, weight and volumes were also significantly suppressed following treatment with curcumin. In conclusion, the investigation demonstrated that curcumin suppressed ACHN cell viability, induced apoptosis and autophagy, through the suppression of AKT/mTOR pathway. Use of curcumin to target AKT/mTOR pathway could be an effective treatment alternative for renal cell carcinoma.

## 1. Introduction

Renal cell carcinoma (RCC) is one of the most lethal cancers, causing significant social and economic burden to humanity [1,2]. Based on statistics, over 300,000 new cases are diagnosed each year with an upward tendency [3]. Unfortunately, the therapies for RCC are highly restrained by the metastatic characteristics and rapid deterioration [4]. Once RCC spreads over the whole body, it hinders the surgical tumor removal and the consequence is poor chemotherapeutic response [5]. According to some clinical reports, intrinsic therapy, such as monoclonal antibodies and kinase inhibitor, could prevent blood vessels from forming in a tumor limiting cancer development [6]. Thus, small molecule drugs targeting to specific signaling pathways while protecting normal cells would show a promising treatment for curing RCC instead of approaching with extrinsic therapeutics.

Previous studies have demonstrated an association between AMP-activated protein kinase (AMPK) signaling and the inhibition of mammalian target of rapamycin (mTOR) cascade, thus suppressing RCC progression [7]. Besides, several mutations including the activation of PIK3, and constitutive reaction of receptor tyrosine kinases, have a role in RCC progression [8]. The PI3K-Akt-mTOR cascade activation increases RCC invasion and reduces therapeutic effects [9,10]. The PI3K-Akt-mTOR signaling over-expression plays an essential role in RCC proliferation, survival, migration, metastasis, angiogenesis, and treatment resistance [10]. Moreover, PI3K-Akt-mTOR signaling is essential for diverse biological process [11]. Previous reports have confirmed the use of mTOR-inhibitors in treatments, hence it can be a promising target for RCC therapy [12].

Curcumin is a traditional Chinese herb that has been used for the treatment of various diseases. The compound has been particularly confirmed to have anti-inflammatory [13], anti-cancer [14,15], anti-virus [16], and anti-oxidant activities [17,18]. Curcumin shows less side effects, hence appropriate as a novel anticancer drug [19]. Previous studies have shown that curcumin administration inhibited the growth, formation, invasion, and proliferation of prostate cancer [18]. However, the role and molecular mechanisms of curcumin in RCC is unclearly defined. The study hypothesized that curcumin induces apoptosis and autophagy through the suppression of AKT/mTOR pathway. The study aimed at assessing whether curcumin induces apoptosis of RCC and the involvement of AKT/mTOR pathway, the effects of curcumin on pro-inflammatory TNF-a, IL6 and IL-8 cytokines, and finally, to understand the role of curcumin in autophagy and tumor-growth in vivo.

## 2. Materials and methods

### 2.1. Provision of reagents

Curcumin (>97.0%) was purchased from TCI (Shanghai, China). The drugs were dissolved in phosphate buffered saline (PBS) and diluted in RPMI-1640 culture medium. RPMI-1640 medium, penicillin-streptomycin, trypsin-EDTA and fetal bovine serum (FBS) were purchased from Gibco (Grand Island, NY, USA). Human ACHN RCC cell line was purchased from the American Type Culture Collection (ATCC, Manassas, VA) and cultured according to the manufacturer’s guidelines.

### 2.2. Cell culture

Cells were cultured with 1640 medium supplemented with 10% FBS and 1% penicillin-streptomycin (50 mg/L penicillin; 100 µg/ml streptomycin) in 75-cm^3^ tissue culture flasks incubating in humidified (5% CO_2_), atmosphere at 37°C. Cells passaging was done at a confluence of 70%.

### 2.3. MTT assay

ACHN cells were seeded into 96-well plates at a density of 1 × 10^5^ cells per well overnight. After cell adherence, various curcumin concentrations (100, 50, 30, 15, 5, 1, 0.1, 0.01, 0.001) µM were introduced into the cells and incubated for 24 h at 37°C. Sterile MTT solution was then added to the treated cells and further incubated for 4 h. Supernatants from each of the wells were gently removed, and the crystals of formazan were dissolved using 150 µl of DMSO, by shaking for 10 min. The absorbance was finally determined at 570 nm using a microplate reader [20]. The curcumin concentration producing 50% inhibition of growth (IC50) was determined through the dose-response curve in Graph Pad Prism (version.5).

### 2.4. ELISA assay

ACHN were incubated with various curcumin concentrations for 24 h. IL-6, IL-8 and TNF-α cytokines were assessed in the ACHN supernatants through Sandwich ELISA using commercial human enzyme-linked immunosorbent assay (ELISA) kits (R&D system, Minneapolis, MN, USA) as per the instructions of the manufacturer. All the appropriate standards and controls were used as specified by the manufacturer [21].

### 2.5. Apoptosis detection

Apoptosis was assessed in a flow cytometer through staining with Annexin V-FITC/propidium iodide (PI). Cells were divided into control, 5, 15, 30, 50 µM curcumin and 3-MA groups then 1 × 106 cells, from every group, were seeded per well in 6-well plates. The cultured cells were then washed twice using ice-cold PBS and stained using Annexin V-FITC and PI using the Apoptosis Detection kit I as per the manufacturer’s instructions (BD Bioscience). Analysis of apoptosis was done using the CellQuest Pro (IVD) software (BD, Bioscience.

### 2.6. The prediction of network pharmacology to explore mechanism of kidney cell cancers treatment by curcumin

To determine the mechanisms of kidney cell cancers treatment by curcumin, the compound was searched on the symmap (http://www.symmap.org/) database. According to the search findings, 108 targets and 150 symptoms were filtered. In this network, the characteristics of kidney cell cancer and targets involved in Akt/mTOR autophagy were screened.

### 2.7. Immunofluorescence assay

ACHN cells were grown on glass slides in culture well plates and then treated with curcumin (5, 15, 30, 50) µM, CQ (1 mM), Z-VAD-FMK (1 mM) or 3-MA (1 mM) and cultured for 24 h. Cells in the culture plate were washed in PBS for three times, fixed with 4% paraformaldehyde for 15 min, and re-washed thrice in PBS. Later, cells were permeabilized using Triton X-100, washed three times in PBS, blocked in 1% BSA, and incubated with rabbit anti-mouse p-AKT antibody (1:200) and LC3B antibody (1:100) overnight. Finally, the samples were incubated with DAPI in the dark for 5 min, and washed in PBST. The slides were dried with an absorbent paper, sealed with a liquid containing anti-fluorescence quenching agent, and finally observed under a fluorescence microscope and the images captured [22].

### 2.8. Cell protein extraction

The culture solution was transferred into a 15-ml centrifuge tube and centrifuged at 12,000 rpm for 5 min, at 4°C. The supernatant was discarded, and then the pellets were re-suspended in 4 mL PBS and centrifuged again for 5 min at 12,000 rpm, and then washed again with PBS. After snap drying, 100 µl of lysis buffer (including PMSF) was added and incubated in ice for 30 min. The lysate was gently mixed, centrifuged at 12000 RPM for 5 min, and the supernatant was divided into 0.5 mL centrifuge tube, and the protein concentration was determined.

### 2.9. Western blot analysis

To determine apoptosis and autophagy, tumor cell lysates were applied to 12% polyacrylamide gel (based on the proteins to be separated) and electrophoresed at 100 V for 90 min. The protein was later transferred to nitrocellulose membranes (100 V, for 1 h). The membrane was then blocked with nonfat dry milk for 1 h, and incubated overnight with monoclonal antibodies directed against t-Akt, p-Akt, mTOR, p-mTOR, LC3B, Beclin-1, p62, cytoc and GAPDH; (1:1000) all: BD Pharmingen). HRP-conjugated goat anti-mouse IgG (1:5.000; Upstate Biotechnology, Lake Placid, NY, USA) was used as the secondary antibody. The membranes were then briefly incubated with ECL detection reagent (ECL; Amersham/GE Healthcare, München, Germany) to detect the proteins and then analyzed by the Fusion FX7 system (Peqlab, Erlangen, Germany). Protein expression was quantified using image J software.

### 2.10. In vivo tumor induction

Healthy 36 C57BL/6 nude mice were acquired from the Animal Experiment Center of Qishan Hospital. All animal experiments were carried out in accordance with the guidelines of the Qishan Hospital Animal Care and Use Committee and the international guidelines on animal use. ACHN cells in the log phase were obtained and suspended in cold PBS to a density of 4 × 106 cells per ml. Next, 100 µl of the suspension of cells was injected through the right posterior flanks of all the experimental mice to obtain growth of tumor. Five days later, mice were randomly divided into 6 groups (n = 6): 0μM Cur group, 15 μM Cur group, 30 μM Cur group, 3-MA+15 μM Cur group, CQ+15 μM Cur group, and Z-VAD+ 15 μM Cur group. Measurements of tumor were determined by a caliper ruler. The tumor volume was determined by the equation: tumor volume (mm^3^) = (length × width^2^)/2. The length and the width were presented in millimeters [23]. After 4 weeks, the mice were euthanized with sodium pentobarbital (100 mg/kg), and the tumor tissues were collected, weighed and stored at −80°C for subsequent biochemical analysis.

### 2.11. Colony formation assay

Approximately, 1 × 10^4^ ACHN cells/well were inoculated on six-well plates for two weeks after treatment with H_2_O_2_, and 5, 15, and 30 μM doses of curcumin. Cell fixation was then done in 10% formaldehyde and subsequently stained in 0.1% crystal violet dye for 15 min. The stained colonies were finally counted in a microscope.

### 2.12. Cell scratch assay

ACHN cells were plated and grown for 24 h at 37°C. A scratch was centrally introduced into the plate with a sterile pipette tip after treatment with H_2_O_2_, and 5, 15, and 30 μM doses of curcumin. Later, wells were carefully washed thrice in PBS, and then a serum-free medium was added. Migration of cells was observed in an inverted microscope per 24 h.

### 2.13. Data analysis

The experimental results were expressed as mean ± standard deviation. Data analysis was done using SPSS 22 software, one-way analysis of variance using Tukey’s test. The difference between the experimental groups was considered significant when p < 0.05.

## 3. Results

### 3.1. Curcumin induce apoptosis of ACHN tumor cells

Renal cell carcinoma is a lethal cancer type, and the treatment remains a challenge. Curcumin is a traditional Chinese medicine with a wide medical use. The current study hypothesized that curcumin induced apoptosis and autophagy through the suppression of AKT/mTOR pathway. The study aimed at assessing whether curcumin induces apoptosis of RCC and the involvement of AKT/mTOR pathway, the effects of curcumin on TNF-α, IL-6 and IL-8 cytokines, and finally, to understand the role of curcumin in autophagy and tumor-growth in vivo.

**Figure 1.**
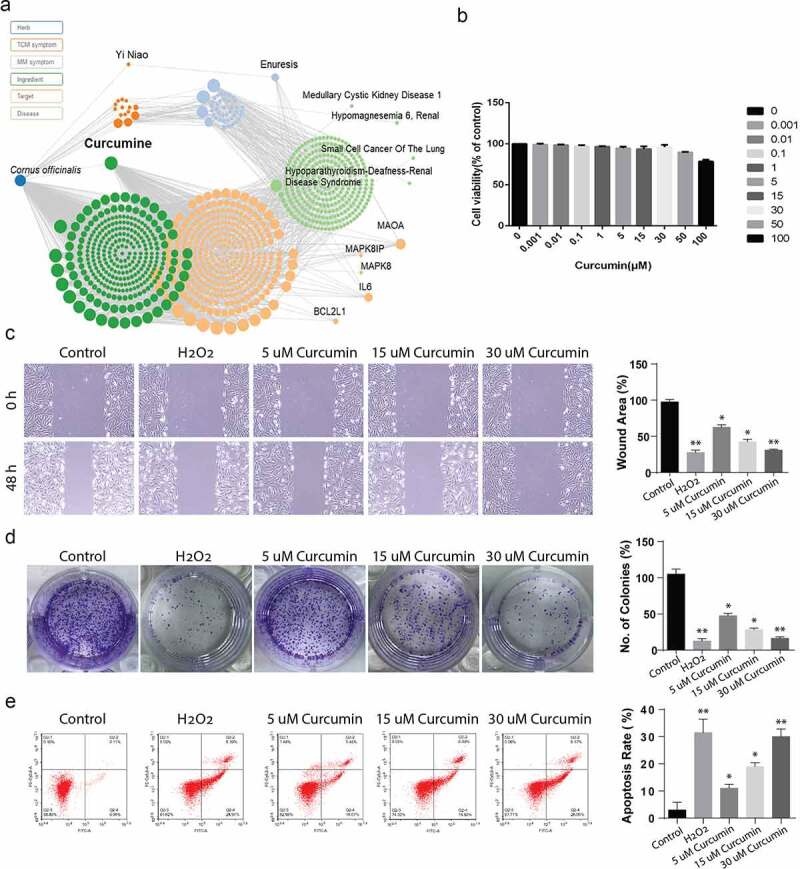
**The network pharmacology prediction of curcumin and toxicology measurement. (**a) The network prediction of the potential targets of curcumin. (b) The toxicology of curcumin was detected by the MTT assay. (c) The wound area (%) of ACHN cells incubated with 5, 15 and 30 µM of curcumin was detected by the scratch assay. (d) The number of colonies (%) of ACHN cells incubated with 5, 15 and 30 µM of curcumin was detected by the colony formation assay. (e) The flow cytometry analysis showing the apoptosis rate of ACHN cells incubated with 5, 15 and 30 µM of curcumin. The value was displayed as the mean ± SD. *P < 0.05, **P < 0.01

According the prediction results obtained from the http://www.symmap.org/, curcumin had a great potential of suppressing renal diseases including hypomagnesemia and multiple cancers (Figure 1(a)). The prediction results in Figure 1(a) are also shown that the curcumin mediated the inflammation targets IL-6 and autophagy targets BCL2L1. Thus, we assumed curcumin may down-regulate renal cell carcinoma. In order to determine suitable curcumin dosages for the experiment, MTT proliferation assays were carried out on ACHN cell lines. According to the results, concentrations from 0.01 µM, 0.1 µM, and 1 µM did not demonstrate any inhibitory effects. However, curcumin concentrations from 5 µM to 100 µm inhibited ACHN cell viability in a dose-dependent manner, as shown in Figure 1(b). Consequently, doses of 5, 15, and 30 μM were picked for use in the subsequent assays. Further, we used scratch assay to evaluate the migration activity after the cell stimulated with 5, 15, and 30 μM doses of curcumin, whereas H_2_O_2_ was used as known apoptosis inducing agent. The result showed that curcumin reduced the cell migration activity in dose-dependent manner (Figure 1(c)). Colony formation assay shows that 5, 15, and 30 μM doses of curcumin reduced the number of colonies in dose-dependent manner (Figure 1(d)). Finally, flow cytometry analysis showed that 5, 15, and 30 μM doses of curcumin induced the cell apoptosis in ACHN cells in dose-dependent manner (Figure 1(e)). Overall, these results shows that curcumin has the potential of inducing apoptosis of ACHN tumor cells.

### 3.2. Curcumin suppresses IL-6, IL-8 and TNF pro-inflammation cytokines

**Figure 2.**
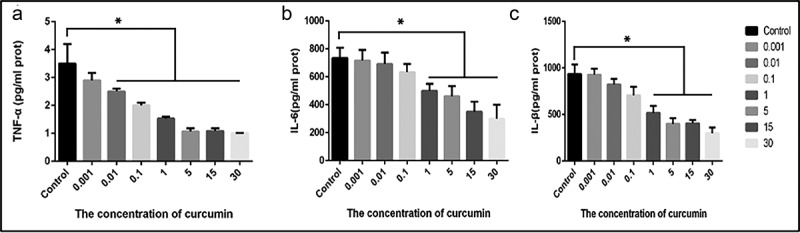
**Curcumin suppresses the IL-6, IL-8 and TNF-a inflammation factors release. (**a) TNF-a, (b) IL-6 and (c) IL-8. The levels of cytokines expressions were determined by ELISA. The values presented are the mean ± SD derived from three independent experiments performed in duplicate and expressed as pg/ mg. The value was displayed as the mean ± SD (n = 6). A one-way analysis of variance was used to calculate the significance of each group, and the variance was corrected using Tukey test. Compared with the Control group, *P < 0.05

Cells, at a population of 1 × 105 per well were plated in the 96-well plates and treated with various curcumin doses of (0.001, 0.01, 0.1, 1, 5, 15 or 30) µM for 24 h. After treatment, the cell suspension was collected and cytokine expression was detected through ELISA. According to the observations, ACHN cells pre-treatment with various curcumin concentrations significantly suppressed the production of TNF-α, IL-6 and IL-8 in a concentration-dependent way in comparison with the control samples, P < 0.05, as shown in Figure 2(a–c). The data show that curcumin have potential to serve as anti-inflammatory target by suppressing pro-inflammatory cytokines.

### 3.3. Curcumin promotes apoptosis in the ACHN cells via AKT/mTOR suppression

**Figure 3.**
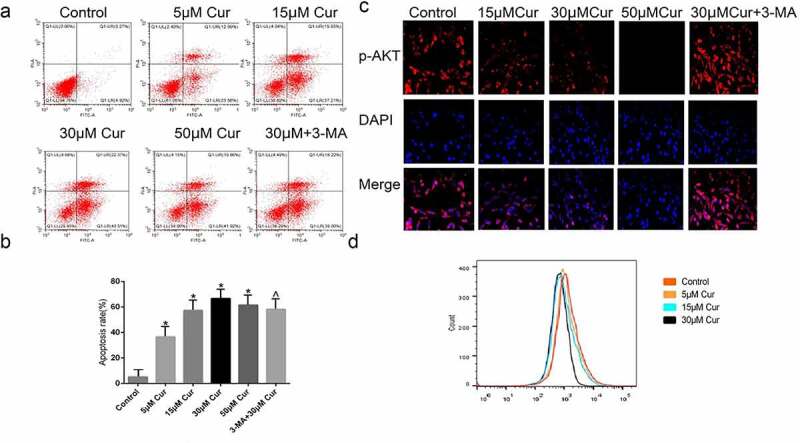
**Curcumin promotes the apoptosis and activates the AKT/mTOR pathway. (**a) Detection of poptosis was done using Annexin V-FITC/PI kit and flow cytometry (b) Graphical representation of quantified apoptosis following treatment with various doses of curcumin or treatment with curcumin in combination with other drugs. (c) Immunofluorescence assay showing the expression of p-AKT following the treatments of ACHN cells with 15 µM curcumin, 30 µM, curcumin, 50 uM curcumin, or 30 µM curcumin+ 3-MA. (d) Quantification of p-AKT immunofluorescence results. The value was displayed as the mean ± SD. A one-way analysis of variance was used to ddetermine the level of significance of each group, in comparison to the control group and the variance was corrected using Tukey’s test.Compared with the Control group, *P < 0.05; Compared with the 30 μM group, ^P < 0.05

To investigate the effects of curcumin on RCC, apoptosis in curcumin-treated ACHN cells was determined through annexin-V FITC/PI. The results demonstrated that 5 μM curcumin led to the apoptosis of 36.92% of ACHN cells, while use of 15 µM curcumin led to apoptosis of 36.92 cells. Likewise, 30 µM and 50 µM produced 65% and 60% of apoptotic cells, respectively. The use of 30 µM curcumin+3-MA produced 57.22% of apoptotic cells, as shown in Figure 3(a,b). Additionally, immunofluorescence staining of ACHN following treatments with curcumin at 15 µM, 30 µM, 50 µM, or with 30 µM+3-MA showed that the expression of p-AKT is inhibited in a dose-dependent manner, from 15 µM to 50 µM. However, treatment with 30 µM+3-MA reversed the down-regulation of p-AKT, as shown in Figure 3(c,d). Taken together, these observations demonstrate that curcumin induce apoptosis by suppressing the AKT/mTOR pathway, and this suppression is reversed by autophagy inhibitor (3-MA). These results show that curcumin promotes apoptosis in the ACHN cell line via AKT/mTOR suppression.

### 3.4. Curcumin down-regulate Akt/mTOR pathway through upregulation of autophagy

**Figure 4.**
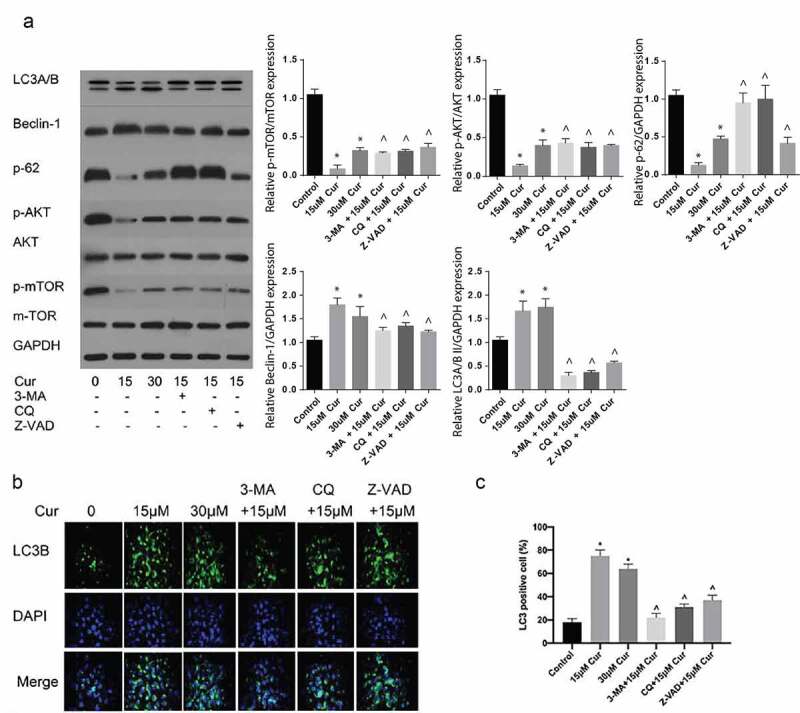
**Curcumin induce autophagy reversed by the autophagy suppressors’ 3-MA and CQ**. (a) Western blot assessment of autophagy related LC3B, Beclin-1 and p62, and AKT/mTOR pathway proteins expressed in ACHN cells following treatment with 15 µM, 30 µM curcumin, 15 µM curcumin+ 3-MA, 15 µM curcumin + CQ or 15 µM curcumin +Z-VAD. (b) Immunofluorescence assessment of LC3B following the various stated treatments was reflected by the immunofluorescence and (c) Graphical quantification of LC3B protein after various treatments. The value was presented as the mean ± SD. A one-way analysis of variance was used to calculate the significance of each group, in comparison to the control and the variance was corrected using Tukey’s test. Compared with the Control group, *P < 0.05 was regarded as significant; Compared with the 15 μM Cur group, ^P < 0.05; GAPDH was used as the internal control

**Figure 5.**
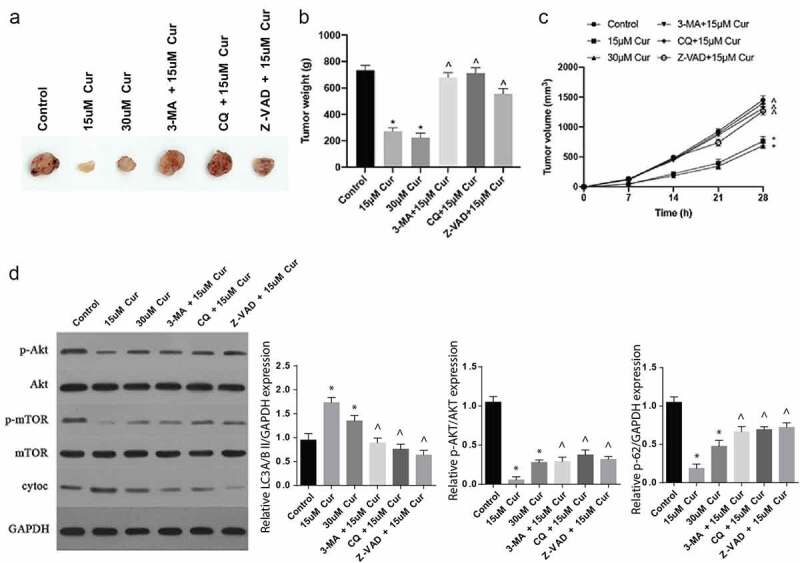
Curcumin inhibits tumor growth in vivo through the Akt/mTOR pathway modulation

To further investigate the role of curcumin in AKT/mTOR and autophagy, ACHN tumor cells were induced in C57BL/6 nude mice and treated with various concentrations of curcumin or 30 µM curcumin +3-MA, 15 µM curcumin + CQ or 15 µM curcumin+ Z-VAD. The mice were sacrificed, and then autophagy and AKT/mTOR-related proteins expressions were determined through western blot. According to the results, treatment with 15 µM or 30 µM of curcumin alone suppressed the p-AKT/mTOR expressions but increased the expressions of autophagy related Beclin 1 and p-62 proteins. However, use of 15 µM curcumin together with 3-MA or CQ reversed the inhibition of p-62, p-AKT or mTOR as shown in Figure 5(a). Further, curcumin together with CQ and 3-MA downregulated the expression of LC3II as compared to the effects of treatment with curcumin alone, as shown in Figure 4(a). Finally, treatment with curcumin +Z-VAD reversed the expression of apoptotic proteins induced by curcumin alone Figure 4(b,c). Taken together, the stated observations confirmed that curcumin initiates apoptosis by inducing autophagy.

### 3.5. Curcumin inhibits tumor growth in vivo through the Akt/mTOR pathway modulation

Finally, the effects of curcumin in RCC were investigated in vivo. Following C57BL/6 nude mice injection with tumor cells and subsequent treatments with various concentrations of curcumin alone, or curcumin with 3-MA, CQ or Z-VAD, various observations were made. The tumor volumes and sizes were significantly reduced following treatments with 15 µm or 30 µM of curcumin, compared to the controls, as shown in Figure 5(a,b). However, treatments with curcumin and 3-MA, CQ or Z-VAD reversed the effect of curcumin treatment alone, thus increased the tumor appearance, weight and size, as shown in Figure 5(a–c). In addition, 15 μM and 30 μM curcumin treatments inhibited tumor p-AKT and p-mTOR expressions and increased cytoc expression. After simultaneous administration of 3-MA, CQ and Z-VAD, the effect of 15 μM curcumin on p-AKT, p-mTOR and cytoc expressions was significantly reversed (Figure 5(d)). Jointly, these observations demonstrate that curcumin regulates the Akt/mTOR signaling pathway through autophagy to inhibit tumor growth.

(a) Photographic images of tumors obtained from mice following tumor induction and treatments with 15 µM, 30 µM curcumin, 15 µM curcumin+ 3-MA, 15 µM curcumin + CQ or 15 µM curcumin +Z-VAD. (b) Graphical representation of tumor weights in grams obtained after various treatments. (c) Line graph representation of tumor volume obtained after various treatments. (d) Western blot results of assessment of Akt/mTOR signaling pathway proteins in mouse tumors following in vivo treatments with 15 µM, 30 µM curcumin, 15 µM curcumin+ 3-MA, 15 µM curcumin + CQ or 15 µM curcumin +Z-VAD; the values were presented as mean ±SD. A one-way analysis of variance was used to calculate the significance of each group, and the variance was corrected using Tukey test. Compared with Control group, *P < 0.05; Compared with 15 μM Cur group, ^P < 0.05.

## 4. Discussion

Even though current treatment approaches against RCC have enhanced the progression-free survival of patients in the advanced stages, this cancer remains ultimately incurable at the advanced stage due to the development of resistance to therapy [24]. Besides, chemotherapy, used in the advanced stages, are also highly associated with resistance and dangerous side effects. Consequently, development of efficient and novel therapy for RCC is quite necessary. The current research investigated the role of curcumin in the treatment of RCC. Interestingly, the study reported that Curcumin has synergistic effects on apoptosis in human ACHN renal carcinoma cells through the modulation of p-AKT/mTOR pathway and promotion of autophagy, in a curcumin-dose dependent manner. Furthermore, combination of curcumin with 3-MA, CQ or Z-VAD reversed the apoptosis and autophagy induced by curcumin.

AKT and mTOR are a well described modulatory signaling pathways that regulates cancer cells survival. Consequently, several inhibitory drugs that block their signaling have been developed and applied for the treatment of tumors. Various studies have reported the use of curcumin in the treatment of different cancers [25], with apoptosis as the mode of tumor cells killing. Further, the use of curcumin in RCC has also been reported [26]. Our findings of a significantly inhibited ACHN cells proliferation is in agreement with a report by Zhang and coworkers who observed the role of curcumin in cell cycle arrest of RCC-949 cell lines [27].

Autophagy is a biochemical process essential in the breakdown of cellular organelles and constituents and often occur as a response to stress, for instance, chemotherapy, environmental toxins, hypoxia and starvation [28], hence plays an essential regulatory role in tumor therapy. Previous studies have shown that autophagy play a vital role in the progression of cancer [29]. The activation of autophagy is highly linked to restriction of tumor genesis and prevention of damage in genome cells [30]. Recent reports stated that cancers, especially breast cancers, are associated with the mutation of autophagy marker, Beclin-1 [31]. Further, multiple studies have shown the inhibition or induction of autophagy by curcumin through the use of specific molecular mechanisms, such as ROS formation and adenosine monophosphate (AMP)-activated protein kinase (AMPK) [32]. The current work reported that curcumin induced the expression of Beclin-1 expression, to confirm induction of autophagy, and autophagy was initiated through the suppression of AKT/mTOR pathway, which is in agreement with the previous reports by Zhang and colleagues [32]. Indeed, from the observation, mTOR seems to act as a negative regulator of autophagy, which had also been reported previously [33]. Furthermore, the current study reports that curcumin promotes the formation of auto-phagosome and upregulated LC3-II, in agreement with the findings [34].

## 5. Conclusion

Curcumin induces apoptosis of RCC through AKT/mTOR pathway inhibition, suppresses the TNF-a, IL6 and IL-8 pro-inflammatory cytokines. Further, curcumin down-regulates Akt/mTOR through upregulation of autophagy and finally, curcumin inhibits tumor weight and volume in vivo. Use of curcumin to target AKT/mTOR pathway could be an effective treatment alternative for renal cell carcinoma.

## Highlights

1: Curcumin inhibits cell viability in the ACHN tumor cells.

2: Curcumin induces the cell apoptosis and autophagy in the ACHN tumor cells.

3: Curcumin suppresses the tumor size, weight, and volumes in-vivo.
